# Mirrored stainless steel substrate provides improved signal for Raman spectroscopy of tissue and cells

**DOI:** 10.1002/jrs.4980

**Published:** 2016-07-29

**Authors:** Aaran T. Lewis, Riana Gaifulina, Martin Isabelle, Jennifer Dorney, Mae L. Woods, Gavin R. Lloyd, Katherine Lau, Manuel Rodriguez‐Justo, Catherine Kendall, Nicholas Stone, Geraint M. Thomas

**Affiliations:** ^1^ Department of Cell and Developmental BiologyUniversity College LondonLondonUK; ^2^ Biophotonics Research UnitGloucester Royal Hospitals NHS Foundation TrustGloucestershireUK; ^3^ School of Physics and Astronomy, College of Engineering, Mathematics and Physical SciencesUniversity of ExeterExeterUK; ^4^ Renishaw PLCNew MillsGloucestershireUK

**Keywords:** Raman spectroscopy, signal improvement, tissue

## Abstract

Raman spectroscopy (RS) is a powerful technique that permits the non‐destructive chemical analysis of cells and tissues without the need for expensive and complex sample preparation. To date, samples have been routinely mounted onto calcium fluoride (CaF_2_) as this material possesses the desired mechanical and optical properties for analysis, but CaF_2_ is both expensive and brittle and this prevents the technique from being routinely adopted. Furthermore, Raman scattering is a weak phenomenon and CaF_2_ provides no means of increasing signal. For RS to be widely adopted, particularly in the clinical field, it is crucial that spectroscopists identify an alternative, low‐cost substrate capable of providing high spectral signal to noise ratios with good spatial resolution. Results show that these desired properties are attainable when using mirrored stainless steel as a Raman substrate. When compared with CaF_2_, data show that stainless steel has a low background signal and provides an average signal increase of 1.43 times during tissue analysis and 1.64 times when analyzing cells. This result is attributed to a double‐pass of the laser beam through the sample where the photons from the source laser and the forward scattered Raman signal are backreflected and retroreflected from the mirrored steel surface and focused towards collection optics. The spatial resolution on stainless steel is at least comparable to that on CaF_2_ and it is not compromised by the reflection of the laser. Steel is a fraction of the cost of CaF_2_ and the reflection and focusing of photons improve signal to noise ratios permitting more rapid mapping. The low cost of steel coupled with its Raman signal increasing properties and robust durability indicates that steel is an ideal substrate for biological and clinical RS as it possesses key advantages over routinely used CaF_2_. © 2016 The Authors. *Journal of Raman Spectroscopy* Published by John Wiley & Sons Ltd.

**Abbreviations**
CaF_2_: Calcium fluoride
RS: Raman spectroscopy
S/N: Signal to noise
STD: Standard deviation

## Introduction

Raman spectroscopy (RS) is a vibrational spectroscopic technique which provides sample‐specific chemical information based on the scattering of photons by molecular bonds. The technique is widely applied in materials science and to biological samples, both *in vivo* and *in vitro*, as water does not significantly interfere with biological signals because the scattering cross section in the fingerprint region, where most peaks of interest reside, is weak. Unlike well‐established methods used to investigate biomolecules, RS requires only minimal sample preparation and does not require the use of dyes as contrast reagents or highly specific molecular probes to provide chemical information relating to both structural and conformational changes. The technique is also minimally invasive, non‐contact and non‐destructive. Recently, there has been a drive to introduce spectroscopic techniques into the clinical diagnostic workflow and a number of studies are currently evaluating the medical capabilities of RS. Raman spectra can be used to generate multivariate classification models capable of objectively identifying discrete tissue pathologies. Studies are being carried out on esophagus^1^, brain^2^, breast^3^, lung^4^ and skin cancers^5^ and a range of other cancers^3^, ^6^.

Despite its many advantages, Raman scattering only occurs for roughly one in every one million photons, which impacts the signal intensity and makes it comparably weaker than other optical techniques. As more chemical information can be extracted from higher signal to noise ratios, various experimental parameters are usually optimized to increase Raman scatter^7^. As the Raman scattering intensity is directly proportional to the intensity of the light source, then high‐powered lasers are most frequently used to improve signal intensities. However, a major drawback to increasing the laser intensity is that some samples may burn under the increased power^8^. The wavelength of the light source, analyte concentration and its sample‐specific scattering properties also affect the signal to noise ratio and are selected accordingly. We implement a 785‐nm laser to reduce fluorescence contribution from tissue, although this results in longer acquisition times in comparison to lower excitation wavelengths because of the inverse‐fourth power dependence of RS with excitation wavelength.

During spectroscopic analysis, samples are routinely mounted on various solid substrates, whose primary role is to support the sample during spectral collection. When selecting a Raman substrate it is important to consider both the physical and optical properties of the material in question as these will affect sample preparation, spectral acquisition and downstream data processing^9^. The background signal of the substrate is material dependent and should be minimized in order to prevent the obscuring of Raman signals by baseline background interference. Appropriate Raman substrates can also be used to significantly enhance signals (surface enhanced Raman scattering) by many orders of magnitude^10^. The most commonly used surfaces for surface enhanced Raman scattering are silver and gold, which exhibit plasmon resonances in the visible to near infrared region, depending on the structure of the nanoscale material^11^. Currently, the most widely used substrate in bio‐Raman is CaF_2_ as the material permits the transmission of visible and near‐IR light with low losses and background absorption, other than the characteristic CaF_2_ peak, which has a Raman shift at 321 cm^−1^ 12. The material also permits transmission FTIR and Raman measurements to be performed on the same sample. Quartz and standard glasses have more intense backgrounds and are more frequently used during SERS and coherent anti Stokes RS setups, where the Raman signal is relatively strong or tuned to a specific wavenumber^12^. Although CaF_2_ provides the desired low background signal and high optical transmission, the material is expensive, many $10s per slide, and brittle. Steel slides are shatterproof and can be obtained for <$3 per slide. The as yet untapped diagnostic potential of RS in conventional pathology laboratory settings motivates the search for a more durable and cost effective substrate. Durability is especially important given the routine use of automated tissue and slide processing machinery in high throughput pathology labs. An ideal substrate should also provide high signal to noise ratios and spatial resolution, if the technique is ever to be adopted in routine clinical practice.

A number of studies have previously investigated the Raman properties of metal and metal‐coated Raman substrates in biological analyses^13^, ^14^ and previous work has demonstrated that polished stainless steel improves spectral intensity and reproducibility and glucose analysis where the laser photons are reflected from the polished metal surface^15^. This observation was also true in azodye analysis by RS^15^. Stainless steel can be manufactured in abundance and at a tiny fraction of the cost of CaF_2_, making it an economically viable replacement for CaF_2_. Unlike CaF_2_, stainless steel is not transparent to white light and must be imaged in reflected‐light mode in order to obtain an image of the sample under investigation. This study examines the optical properties of mirrored stainless steel in Raman analysis and aims to provide evidence to encourage the use of mirrored (highly polished) stainless steel as a Raman substrate for bio‐Raman applications.

## Methods

### Stainless steel substrate preparation

Grade 304 super mirror stainless steel (UNS S30400) of 0.9‐mm thickness with a bright buffed finish was laser cut to standard microscope slide dimensions (75 mm × 24 mm × 0.9 mm) were provided by Renishaw PLC (Gloucester, UK). Slides were cleaned by sonication in trichloroethylene for 30 min, followed by acetone for 30 min and finally, isopropanol for a further 30 min. Slides were then dried under a stream of nitrogen and stored at room temperature.

### Tissue preparation and measurement

Formalin fixed paraffin embedded colon tissue blocks were obtained from the UCL Biobank (REC 15/YH/0311). For each tissue block contiguous 8‐µm tissue sections were prepared for RS on stainless steel and Raman grade CaF_2_ slides of dimension 76 × 26 × 1 mm (Crystran, Poole, UK). Three‐micrometer tissue sections were also cut and hematoxylin and eosin stained for histological review by a consultant histopathologist. All samples were dewaxed with xylene with four, 5‐min baths prior to downstream analysis. Dewaxed samples were stored at room temperature and comparable tissue sections were Raman mapped on the same day.

A Renishaw RA800 series benchtop Raman system configured for pathology use; 1200 l/mm grating, 785‐nm excitation wavelength with a ×50, NA 0.75 objective, motorized XYZ stage was used for all measurements. The system is equipped with transmitted and reflected white light imaging for sample location. StreamLine™ Raman imaging, a fast mapping method, was used for data collection. The spectral resolution was 2.5 cm^−1^ with a range of 150–2100 cm^−1^. Fully automated alignment and calibration routines ensure data reproducibility and transferability. Spectral collection is controlled by WiRE 4.0 software (Renishaw PLC). Raman maps were collected from identical tissue regions at an acquisition time of 0.3 s per pixel at 1.05‐µm spatial resolution. A 785‐nm laser was used for measurements with a laser power of 230 mW measured at the sample. A total of 262 208 spectra from 20 tissue maps were analyzed.

### Cell culture and measurement

U‐2 OS (human bone osteosarcoma) cells were grown in McCoy's 5a medium, supplemented with 10% fetal bovine serum. Cells were seeded overnight onto 20 mm × 1 mm Raman‐grade CaF_2_ discs (Crystran, Poole, UK) and stainless steel disks of comparable dimensions in six‐well culture plates. Cells were washed with phosphate‐buffered saline and air dried prior to Raman mapping. Individual cells of similar size and morphology were isolated on both substrates for comparison and a total of 2000 spectra from 20 individual cell maps were analyzed.

### Data processing and analysis

Streamline maps were loaded into Matlab R2014b (The Mathworks Inc., Natick, Massachusetts, USA) for data pre‐processing. The spectral range was cropped to the fingerprint region 450–1800 cm^−1^. Cosmic rays and fluorescence saturated pixels were filtered from the spectral data by applying a 5 × 5 moving window, two‐dimensional median filter to each wavenumber. For the 5 × 5 window used, the center pixel was replaced with the median value of all pixels in the window for the chosen wavenumber. Signal to noise (S/N) ratios were calculated for the phenylalanine ring breathing mode at 1003 cm^−1^ and the CH_2_/CH_3_ stretching mode at 1450 cm^−1^. S/N were calculated by subtracting the background photon count at 1784 cm^−1^ from to total photon counts for each peak and diving this by the square root of the background photon count.

### Polystyrene measurements

In order to ascertain whether there were any differences in spatial resolution between CaF_2_ and steel, polystyrene microspheres of 90‐µm diameter (coefficient of variance = 10%) and 4.5‐µm diameter (coefficient of variance = 7%) (Polysciences, EU, GmbH) were Raman mapped on both CaF_2_ and stainless steel using the tissue measurement parameters. Individual polystyrene microspheres were suspended in distilled water and isolated under a microscope, spotted onto slides and air dried prior to measurement. Polystyrene coverage was measured by mapping the intensity of the polystyrene‐associated Raman shift at 1002 cm^−1^. Raman intensities falling within 5–95% range were converted to a binary area and the total pixel number calculated using the area measurement function in ImageJ^16^.

## Results and discussion

Two major barriers to the widespread implementation of RS are its slow mapping speed in comparison to other optical techniques, such as fluorescence microscopy, and the expense associated with CaF_2_, the most commonly used Raman substrate. A number of substrates with the potential of overcoming these obstacles have been characterized by our group (unpublished data) and others^13^, ^17^, including synthetic fused silica, aluminum coated glass, borosilicate glass and extra white soda lime glass (unpublished data). Here we present the Raman performance of mirrored stainless steel, which was experimentally determined as the most suitable replacement for CaF_2_ for use as a Raman substrate.

### Raman background measurements of mirrored stainless steel

**Figure 1:**
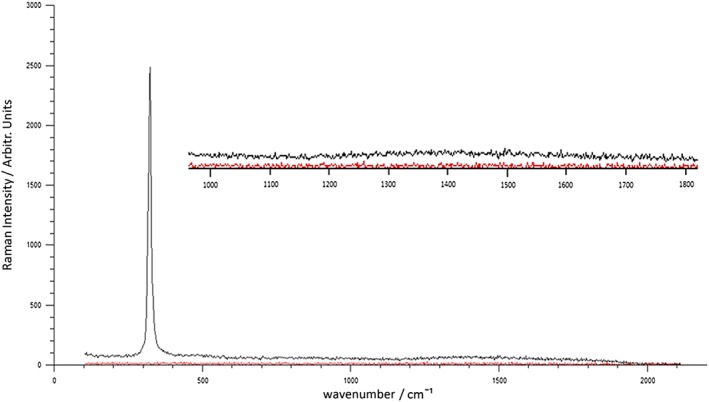
Raman background spectra of calcium fluoride (Black) and Steel (Red) with spectral region between 1000 and 1800 cm^−1^ enlarged.

First, in order to determine the Raman background signal of mirrored stainless steel in comparison to CaF_2_, five point measurements were taken across five steel and five CaF_2_ slides. A measurement was taken at the center of each slide and at each of the slides four corners in order to obtain good coverage. Representative spectra of steel and CaF_2_ can be seen in (Fig. 1). It is advantageous for the spectral contribution of the substrate to be as low as possible in order to minimize the obscuring of Raman tissue signals, which negates the need for extensive downstream processing that could lead to losses of the true signal^18^. The maximum background signal for steel is lower by a factor of six‐fold than CaF_2_ across the entire fingerprint region and has no Raman active modes in comparison to the well‐characterized, single CaF_2_ peak at 321 cm^−1^. As metals are infinitely polarizable and the electron–phonon coupling is very low we would not expect a signal from stainless steel as it does not fulfill the requirements for Raman scattering. Based on the low baseline Raman intensity of steel it seems that the substrate will not obscure Raman peaks arising from tissue scatter and that the use of substrate background subtraction methods is likely to be minimized or made redundant.

### Tissue and cell Raman signal improvements

**Figure 2:**
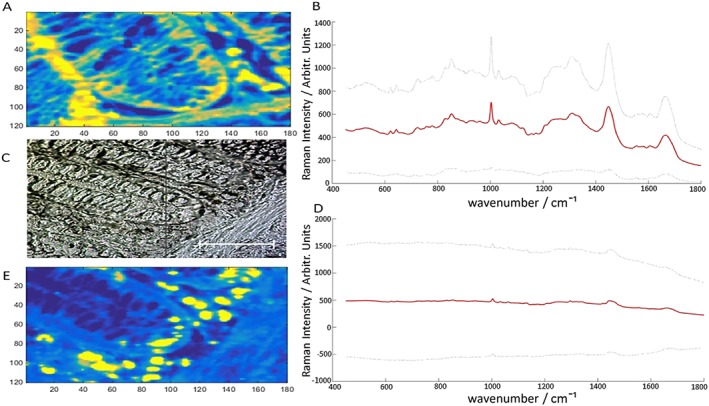
Raman map showing the intensity of the Raman shift at 1002 cm^−1^ for tissue on steel (A) with corresponding mean Raman spectrum with two standard deviations (B). White light image of Raman mapped crypt region (C). Mean Raman spectrum of tissue on CaF2 with two standard deviations. Scale bar is 50 µm (D). Raman map showing the intensity of the Raman shift at 1002 cm^−1^ for tissue on CaF2 (E).

**Figure 3:**
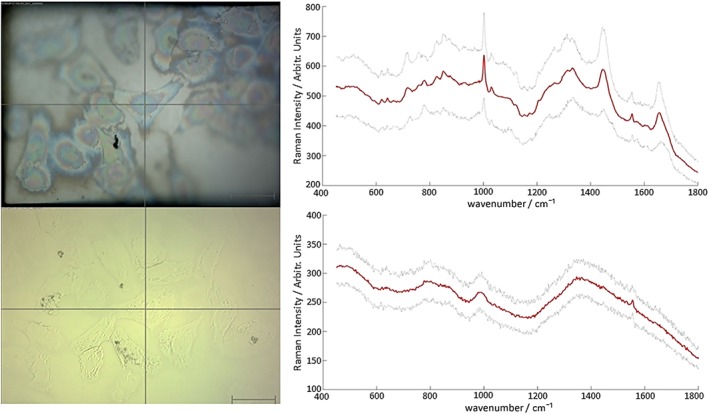
Upper panels show the median Raman spectrum from a cell map on steel (red line) along with error shown as a sleeve of two standard deviations (grey lines, top right) and corresponding white light image (top left). Lower panels show the median Raman spectrum from a cell map on CaF2 (red line) with error shown as a sleeve of two standard deviations (grey lines, lower right) with corresponding white light image (lower left). Scale bars represent 50 µm.

Identically sized Raman maps were generated from comparable regions of contiguous colon tissue mounted on mirrored stainless steel and CaF_2._ Raman maps ranged from 180 × 120 pixels to 50 × 50 pixels for each sample depending on the organelle under investigation. Larger maps were used to cover individual colonic crypts and smaller maps were generated on homogeneous regions of tissue, including regions of muscle and fatty tissue. An averaged spectrum from a Raman map generated from a crypt region on CaF_2_ and mirrored stainless steel can be seen in (Fig. 2). The most prominent Raman peaks in the spectra occur at Amide I (1620 – 1680 cm^−1^) and Amide III (1220 – 1270 cm^−1^) and are attributed to protein backbone vibrations^19^. Another intense band centered around 1002 cm^−1^, attributed to phenylalanine, can be seen in the spectra and is a stable measure of protein distribution in cells^20^. Various other vibrational modes attributed to amino acids, carbohydrates and nucleotides occur throughout the spectrum between 650 cm^−1^ and 1600 cm^−1^ 21. Raman maps were also generated from U‐2 OS cells on mirrored stainless steel and CaF_2_ (Fig. 3)_._

**Figure 4:**
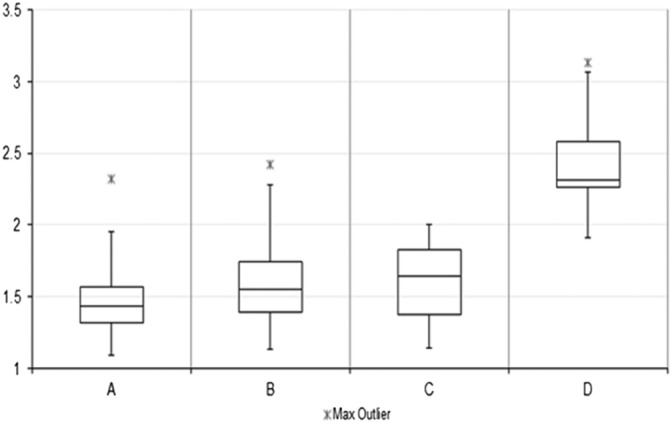
Box plots showing the distribution of magnitudes of Raman signal increases for colon tissue on steel using phenylalanine peak at 1003 cm^−1^ (A) and CH2/CH3 stretching mode at 1450 cm^−1^ (B). Raman signal increases for U2‐OS cells on steel using phenylalanine peak at 1003 cm^−1^ (C) and CH2/CH3 stretching mode at 1450 cm^−1^.

For tissue, calculated S/N ratios are in the range of 15.30 – 239.11 and are notably higher on all samples measured on stainless steel. See Tables S1–S4 (Supporting Information) for full S/N ratios. To easily convey the increases in Raman signals, a Raman improvement factor was calculated by dividing the total signal to noise on mirrored stainless steel by the total S/N ratio on CaF_2_ for each identical mapped region. For the phenylalanine ring breathing mode at 1003 cm^−1^, a median Raman improvement factor of 1.43 was calculated for tissue, with a maximum of 2.30 and a minimum of 1.09 (Fig. 4). For the CH_2_/CH_3_ stretching mode at 1450 cm^−1^, a median Raman improvement factor of 1.55 was calculated, with a maximum of 2.42 and a minimum of 1.13 (Fig. 4). Cell maps spanned both the nuclear and cytoplasmic regions of cells in order to capture representative spectra covering the full range of cellular components. Signal to noise ratios for cell spectra ranged from 77.43 to 175.36, with a median Raman improvement factor of 1.64 for the phenylalanine peak. S/N ratios ranged between 8.2 and 26.9 for the peak at 1450 cm^−1^ and the median Raman improvement calculated to be 2.32. Cells readily attached to both stainless steel and CaF_2_ over the same time period and normal morphology was retained, suggesting that the cells grow equally well on both substrates. Furthermore, we have cultured this cell line on multiple pieces of steel and calcium fluoride over a period of weeks and they display normal morphology and adherence. Cells also reach sampling confluence of 80% on both steel and CaF_2_ within the same time period, suggesting that they grow equally well on both substrates. Cells absorb very little white light providing a very limited contrast in their native state. However, from the white light images it can be seen that the phase contrast of cells on stainless steel, in reflected mode, is greater when compared to cells on CaF_2_. Cell nuclei and cytoplasmic and nuclear boundaries can be easily distinguished on stainless steel permitting more accurate measurement of these cellular compartments without the need for dyes and contrast agents and improved phase contrast on steel alone could prove useful for researchers and clinicians who endeavor to view and measure cells in their natural state.

The S/N ratios for both tissue and cells measured on stainless steel were consistently higher than that for comparable samples measured on CaF_2._ The S/N ratio is arguably one of the most important spectral characteristics and determines the usefulness of the obtained spectrum^22^. The improved S/N ratios obtained on steel permit a greater number of spectral features to be identified and will likely improve spectral acquisition in thin tissue sections, particularly where tissue coverage is sparse and it may be difficult to achieve a Raman signal on standard substrate. Increased Raman signal is attainable at lower acquisition times on steel, leading to a reduction in mapping time and it is likely that maps could be generated more rapidly without compromising on signal quality.

The improvement in Raman signal on stainless steel is likely attributed to a double‐pass of the laser beam through the tissue^15^, ^23^. During the double‐pass, the laser photons are reflected by the steel surface and are redirected back through the tissue where they are presented with a second opportunity to be Raman scattered, leading to an increase in the total number of Raman scattered photons available for detection. The steel could also reflect back and concentrate a higher proportion of Raman photons towards collection optics. However, it is predicted that signal improvements because of the double‐pass will only be observable in transparent and thin samples, where the laser focus is at the interface, as these increases would likely be lost in thick and opaque samples because of the retroreflected laser not being efficiently collected and collimated by the objective. This hypothesis is in line with the observed magnitude of signal improvement of 1.43 times for tissue and 2.31 times for cell measurements as we would expect an increase of between 1.01 and 2.00 times if the laser photons were predicted to experience a second encounter with tissue matter, with additional increases because of the focusing of scattered photons towards collection optics. Any measurements outside of this range could also be caused by variations in tissue thickness between sections. Furthermore, all measurements for cell maps were within this expected range as we would not expect as large a variation in sample thickness in a cell monolayer^24^.

### Polystyrene

**Figure 5:**
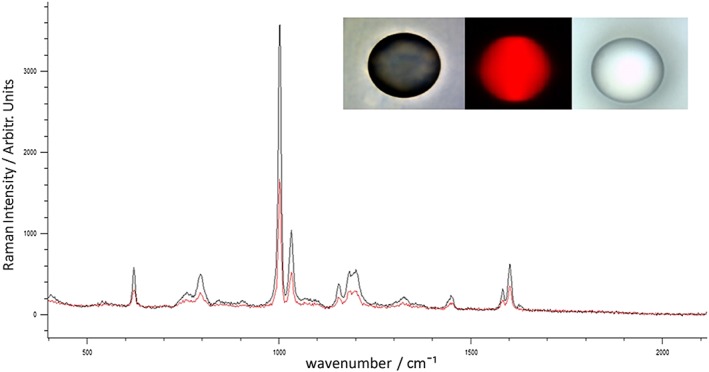
Raman spectra and signal distributions of a 90‐µm polystyrene microsphere on calcium fluoride (red spectrum) and steel (black spectrum) with white light image of polystyrene ball on CaF2 (right image), Raman signal distribution (middle image) and white light image of polystyrene ball on steel (left image).

To ensure that the spatial resolution was not compromised on mirrored stainless steel substrate, polystyrene microspheres of 90‐µm diameter were mapped on stainless steel and CaF_2_. White light images and the Raman scattering signal at 1002 cm^−1^ for polystyrene microspheres on steel and CaF_2_ can be seen in Fig. 5.

The calculated areas and x, y dimensions of six 90‐µm and 4.5‐µm polystyrene microspheres on both substrates can be viewed in Tables 1 and 2 respectively. The calculated maximum cross‐sectional area of polystyrene balls, when treated as a two dimension circle as detectable by Raman, is given by the equation *A* = *πr* ^2^. For the larger microspheres, when using the radius of 45 µm attributed to a 90‐µm diameter circle the total area detectable by Raman is 6362 µm^2^, which is in line with the Raman data when considering some pixel loss at the circumference because of signal thresholding. For the smaller spheres, we would expect an area of 22 µm^2^. There was no significant difference between the spatial resolution on stainless steel and CaF_2_. The average pixel areas calculated for both substrates were in the expected range and within the manufacturers quoted variability, where the average area of polystyrene on steel was calculated at 6260 µm^2^ and at 6090 µm^2^ on CaF_2_ for the 90‐µm spheres and 20 µm^2^ *versus* 23 µm^2^ when using the 4.5‐µm spheres. The calculated Raman area was closer to the expected area on stainless steel substrate and was also less variable on steel; the standard deviations in the calculated polystyrene areas were significantly smaller on stainless steel, supporting, at minimum, a comparable spatial resolution on stainless steel when taking sphere variability into account.

**Table 1:** Calculated Raman areas and x,y measurements of 90‐µm polystyrene microspheres on steel and calcium fluoride with average area and standard deviations for each substrate

	Polystyrene area/µm^2^		Steel x, y measurements/µm		CaF_2_ x, y measurements/µm	
Measurement	Steel	CaF_2_	x	y	x	y
1	6285	6471	89	88	87	94
2	6272	5681	87	90	82	88
3	6266	6674	87	89	94	92
4	6124	6043	86	89	86	89
5	6316	5898	85	89	83	89
6	6296	5775	89	88	82	88
Average	6260	6090	87	89	86	90
Standard deviation	63	363	2	1	5	2

**Table 2:** Calculated Raman areas and x,y measurements of 4.5‐µm polystyrene microspheres on steel and calcium fluoride with average area and standard deviations for each substrate

	Polystyrene area/µm^2^		Steel x, y measurements/µm		CaF_2_ x, y measurements/µm	
Measurement	Steel	CaF_2_	x	y	x	y
1	20	21	4	3	4	4
2	20	24	4	4	5	4
3	19	26	4	4	5	5
4	23	18	4	4	4	4
5	22	25	4	4	4	4
6	18	21	4	4	4	4
Average	20	23	4	4	4	4
Standard deviation	2	3	0	0	1	0

## Conclusion

This work has assessed the Raman optical properties of mirrored stainless steel in the analysis of both tissues and cells. Our data verifies the suitability of stainless steel as a Raman substrate for the analysis of biological tissue using human colon sections and human cells. Clear and intense increases in signal were observed on stainless steel when analyzing both tissues and cells. Data show that the improvements do not have a detrimental effect on spatial resolution because of an additional reflection of the laser beam on the steel surface when polystyrene microspheres of defined dimensions were analyzed on both substrates. Results showed that spatial resolution was closer to the calculated value on stainless steel and that the standard deviation between measurements was also considerably lower on steel. Therefore, it is unlikely that the detected spatial distribution of biological components would be compromised when mounted on steel for RS.

In summary, stainless steel improves Raman signal and permits a more rapid spectral acquisition without compromising spatial resolution. Lower acquisition times would reduce localized heating and tissue degradation and this work provides evidence to support the use of mirrored stainless steel for Raman spectroscopic measurements of cells and tissues with the hope that steel will be more widely implemented in the field. Furthermore, mirrored stainless steel can provide a much more cost‐effective and durable substrate capable of supporting the high throughput analysis needed to implement RS in the routine clinical field, a field that is highly unlikely to implement CaF_2_ because of its excessive cost and the degree of care required in handling which will confound automation^25^. We hope that our data will persuade more spectroscopists to utilize steel more frequently in their bio‐Raman applications. Furthermore, as part of ongoing studies into the routine application RS in clinical diagnosis we are currently building discriminatory models for both colorectal and esophageal cancers on stainless steel substrate. These proof of principle models, along with our recent advances in minimizing inter‐instrument system variability,^26^ underpin a thorough assessment of the clinical potential of this very promising material.

## Author contributions

Dr Aaran T. Lewis and Miss Riana Gaifulina contributed equally to this work. All authors have given approval to the final version of this manuscript.

## Supporting information

Table S1 Signal to noise ratios and Raman signal increases in tissue *for phenylalanine ring breathing mode at 1003 cm* ^−*1*^ on calcium fluoride and steel
Table S2 Signal to noise ratios and Raman signal increases in tissue *for CH_2_*/*CH_3_ stretching modes at 1450 cm* ^−*1*^ on calcium fluoride and steel
Table S3 Signal to noise ratios and Raman signal increases for cells *for phenylalanine ring breathing mode at 1003 cm* ^−*1*^ on calcium fluoride and steel
Table S4 Signal to noise ratios and Raman signal increase for cells *for CH_2_*/*CH_3_ stretching modes at 1450 cm* ^−*1*^ on calcium fluoride and steel

Supporting info item
