# Supplementary material for: Mirrored stainless steel substrate provides improved signal for Raman spectroscopy of tissue and cells
Source: J Raman Spectrosc. 2016 Jul 29;48(1):119–25. doi: 10.1002/jrs.4980 (PMC5256423; doi:10.1002/jrs.4980)
Supplement: Supplementary file 1 — Supporting info item [file JRS-48-119-s001.docx]

**Supporting Information**

Table S1 Signal to noise ratios and Raman signal increases in tissue for phenylalanine ring breathing mode at 1003 cm^-1^ on calcium fluoride and steel

| Sample | Calcium Fluoride | Steel | Raman Signal Increase |
| --- | --- | --- | --- |
| 1 | 123.36 | 158.09 | 1.28 |
| 2 | 152.39 | 225.10 | 1.48 |
| 3 | 148.23 | 213.72 | 1.44 |
| 4 | 166.01 | 181.70 | 1.09 |
| 5 | 121.38 | 169.71 | 1.40 |
| 6 | 129.67 | 226.07 | 1.74 |
| 7 | 96.62 | 151.49 | 1.57 |
| 8 | 141.89 | 231.11 | 1.63 |
| 9 | 138.30 | 216.57 | 1.57 |
| 10 | 150.56 | 176.64 | 1.17 |
| 11 | 168.68 | 239.11 | 1.42 |
| 12 | 150.56 | 251.75 | 1.67 |
| 13 | 154.13 | 203.40 | 1.32 |
| 14 | 134.90 | 159.20 | 1.18 |
| 15 | 92.16 | 123.80 | 1.34 |
| 16 | 153.60 | 222.17 | 1.45 |
| 17 | 96.40 | 223.99 | 2.32 |
| 18 | 153.66 | 232.95 | 1.52 |
| 19 | 135.02 | 175.92 | 1.30 |
| 20 | 138.78 | 192.08 | 1.38 |

Table S2 Signal to noise ratios and Raman signal increases in tissue for CH_2_/CH_3_ stretching modes at 1450 cm^-1^ on calcium fluoride and steel

| Sample | Calcium Fluoride | Steel | Raman Signal Increase |
| --- | --- | --- | --- |
| 1 | 19.6 | 27.7 | 1.41 |
| 2 | 20.4 | 33.4 | 1.64 |
| 3 | 25.0 | 43.6 | 1.74 |
| 4 | 36.8 | 48.6 | 1.32 |
| 5 | 19.1 | 26.0 | 1.36 |
| 6 | 18.6 | 45.1 | 2.42 |
| 7 | 14.1 | 25.9 | 1.84 |
| 8 | 19.3 | 31.5 | 1.63 |
| 9 | 22.0 | 31.1 | 1.41 |
| 10 | 20.6 | 32.0 | 1.55 |
| 11 | 22.1 | 39.2 | 1.77 |
| 12 | 17.6 | 24.6 | 1.40 |
| 13 | 20.8 | 36.2 | 1.74 |
| 14 | 19.4 | 38.8 | 2.00 |
| 15 | 21.3 | 33.1 | 1.55 |
| 16 | 18.0 | 23.9 | 1.33 |
| 17 | 15.3 | 33.1 | 2.16 |
| 18 | 18.0 | 23.8 | 1.32 |
| 19 | 15.3 | 17.3 | 1.13 |
| 20 | 22.9 | 35.4 | 1.55 |

Table S3 Signal to noise ratios and Raman signal increases for cells for phenylalanine ring breathing mode at 1003 cm^-1^ on calcium fluoride and steel

| Sample | Calcium Fluoride | Steel | Raman Signal Increase |
| --- | --- | --- | --- |
| 1 | 82.52 | 93.85 | 1.14 |
| 2 | 88.62 | 149.64 | 1.69 |
| 3 | 89.55 | 108.55 | 1.21 |
| 4 | 84.48 | 114.30 | 1.35 |
| 5 | 82.32 | 131.60 | 1.60 |
| 6 | 84.83 | 146.90 | 1.73 |
| 7 | 77.43 | 143.81 | 1.86 |
| 8 | 83.94 | 120.56 | 1.44 |
| 9 | 87.72 | 175.36 | 2.00 |
| 10 | 85.93 | 165.80 | 1.93 |

Table S4 Signal to noise ratios and Raman signal increase for cells for CH_2_/CH_3_ stretching modes at 1450 cm^-1^ on calcium fluoride and steel

| Sample | Calcium Fluoride | Steel | Raman Signal Increase |
| --- | --- | --- | --- |
| 1 | 19.0 | 8.3 | 2.29 |
| 2 | 23.4 | 8.8 | 2.66 |
| 3 | 19.4 | 8.3 | 2.34 |
| 4 | 16.8 | 8.8 | 1.91 |
| 5 | 17.8 | 8.2 | 2.17 |
| 6 | 18.5 | 8.2 | 2.26 |
| 7 | 20.0 | 8.5 | 2.35 |
| 8 | 19.0 | 8.4 | 2.26 |
| 9 | 26.9 | 8.6 | 3.13 |
| 10 | 23.8 | 8.3 | 2.87 |
